# A Digital Intervention for Adolescent Depression (MoodHwb): Mixed Methods Feasibility Evaluation

**DOI:** 10.2196/14536

**Published:** 2020-07-17

**Authors:** Rhys Bevan Jones, Anita Thapar, Frances Rice, Becky Mars, Sharifah Shameem Agha, Daniel Smith, Sally Merry, Paul Stallard, Ajay K Thapar, Ian Jones, Sharon A Simpson

**Affiliations:** 1 Division of Psychological Medicine and Clinical Neurosciences Medical Research Council Centre for Neuropsychiatric Genetics and Genomics Cardiff University Cardiff, Wales United Kingdom; 2 National Centre for Mental Health Cardiff University Cardiff, Wales United Kingdom; 3 Cwm Taf Morgannwg University Health Board Wales United Kingdom; 4 Population Health Sciences University of Bristol Bristol, England United Kingdom; 5 Institute of Health and Wellbeing University of Glasgow Glasgow, Scotland United Kingdom; 6 Faculty of Medical and Health Sciences School of Medicine University of Auckland Auckland New Zealand; 7 Department for Health University of Bath Bath, England United Kingdom; 8 Medical Research Council/Chief Scientist Office Social and Public Health Sciences Unit Institute of Health and Wellbeing University of Glasgow Glasgow, Scotland United Kingdom

**Keywords:** adolescent, depression, internet, education, early medical intervention, feasibility study

## Abstract

**Background:**

Treatment and prevention guidelines highlight the key role of health information and evidence-based psychosocial interventions for adolescent depression. Digital health technologies and psychoeducational interventions have been recommended to help engage young people and to provide accurate health information, enhance self-management skills, and promote social support. However, few digital psychoeducational interventions for adolescent depression have been robustly developed and evaluated in line with research guidance.

**Objective:**

We aimed to evaluate the feasibility, acceptability, and potential impact of a theory-informed, co-designed digital intervention program, MoodHwb.

**Methods:**

We used a mixed methods (quantitative and qualitative) approach to evaluate the program and the assessment process. Adolescents with or at elevated risk of depression and their parents and carers were recruited from mental health services, school counselors and nurses, and participants from a previous study. They completed a range of questionnaires before and after the program (related to the feasibility and acceptability of the program and evaluation process, and changes in mood, knowledge, attitudes, and behavior), and their Web usage was monitored. A subsample was also interviewed. A focus group was conducted with professionals from health, education, social, and youth services and charities. Interview and focus group transcripts were analyzed using thematic analysis with NVivo 10 (QSR International Pty Ltd).

**Results:**

A total of 44 young people and 31 parents or carers were recruited, of which 36 (82%) young people and 21 (68%) parents or carers completed follow-up questionnaires. In all, 19 young people and 12 parents or carers were interviewed. Overall, 13 professionals from a range of disciplines participated in the focus group. The key themes from the interviews and groups related to the design features, sections and content, and integration and context of the program in the young person’s life. Overall, the participants found the intervention engaging, clear, user-friendly, and comprehensive, and stated that it could be integrated into existing services. Young people found the “Self help” section and “Mood monitor” particularly helpful. The findings provided initial support for the intervention program theory, for example, depression literacy improved after using the intervention (difference in mean literacy score: 1.7, 95% CI 0.8 to 2.6; *P*<.001 for young people; 1.3, 95% CI 0.4 to 2.2; *P*=.006 for parents and carers).

**Conclusions:**

Findings from this early stage evaluation suggest that MoodHwb and the assessment process were feasible and acceptable, and that the intervention has the potential to be helpful for young people, families and carers as an early intervention program in health, education, social, and youth services and charities. A randomized controlled trial is needed to further evaluate the digital program.

## Introduction

### Prevention and Management of Adolescent Depression

Adolescent depression is common and is associated with social and educational impairments, deliberate self-harm, and suicide. It can also mark the beginning of long-term mental health difficulties [1]. Early treatment and prevention of adolescent depression is therefore a major public health concern [2]. However, many adolescents with depression do not access interventions, and engaging young people in prevention and early intervention programs is challenging [1,3,4].

Guidelines for the prevention and management of depression in young people [5,6] stress the need for good information and evidence-based psychosocial interventions for the young person, family, and carer. There has been growing interest in psychoeducational interventions, which deliver accurate information to individuals, families, and carers about mental health or a specific diagnosis, management and prognosis, and relapse prevention strategies [6-8]. The American Academy of Child and Adolescent Psychiatry (2007) “Practice parameter for the assessment and treatment of children and adolescents with depressive disorders” describes psychoeducation as the “education of family members and the patient about the causes, symptoms, course, and different treatments of depression and the risks associated with these treatments as well as no treatment at all. Education should make the treatment and decision-making process transparent and should enlist parent and patient as collaborators in their own care.” [6] Although the risk factors and possible causes of adolescent depression are complex, a family history of depression, psychosocial stress, and a previous history of depression increase individual risk, and these groups could be targeted for such strategies [9].

Findings from a systematic review concluded that psychoeducational interventions were effective in improving the clinical course, treatment adherence, and psychosocial functioning of adults with depression [10]. However, a systematic review of such interventions for adolescent depression [11] showed that there were few existing programs. This is an important gap in the literature because depression is common in young people, and its presentation and management are different from those of adults [1].

### Digital Mental Health

Digital health or electronic health (eHealth) has been identified as a key area of future clinical practice and research in adolescent depression, especially to improve reach and access to therapies at relatively low cost [8,12]. There is evidence to support the use of some digital interventions for adolescent depression, and they have been recommended in treatment and prevention guidelines [5,13-16]. However, to our knowledge, there is no digital psychoeducational intervention that has been co-designed and specifically developed for adolescents with depression, or those at elevated risk, or developed and evaluated in line with key guidance of digital and complex interventions [17-20].

Here, we describe an early stage evaluation of MoodHwb, a digital psychosocial intervention co-designed with, and for young people with, or at elevated risk of depression, and their families and carers [21,22]. The main aim of this analysis was to examine whether the program and assessments were feasible and acceptable. This is a crucial step to support the refinement of the prototype and design of a randomized controlled trial [20]. This work could also help to inform the development and evaluation of other digital technologies in this field.

## Methods

### The Digital Intervention: MoodHwb

MoodHwb was designed to engage young people (and families and carers) by using developmentally appropriate language, illustrations, animations, and interactive components. It also aims to promote self help, help-seeking where appropriate, and social support. Although it was founded primarily on psychoeducation, it also includes elements of cognitive behavioral therapy, positive psychology, and interpersonal, family systems and behavioral change theories. It is multi-platform and is available as an app, which includes the interactive components and a mobile-friendly version of the main site.

The program was co-designed using a series of interviews and focus groups with potential users: young people (with depressive symptoms or at high risk), parents, carers, and professionals from health, education, social, and youth services and charities. It was also informed by the systematic review noted earlier [11]; person-centered guidelines for developing digital interventions [17-19]; design, educational, and psychological theory; a logic model; and consultations with a digital media company and clinical/research experts.

**Figure 1:**
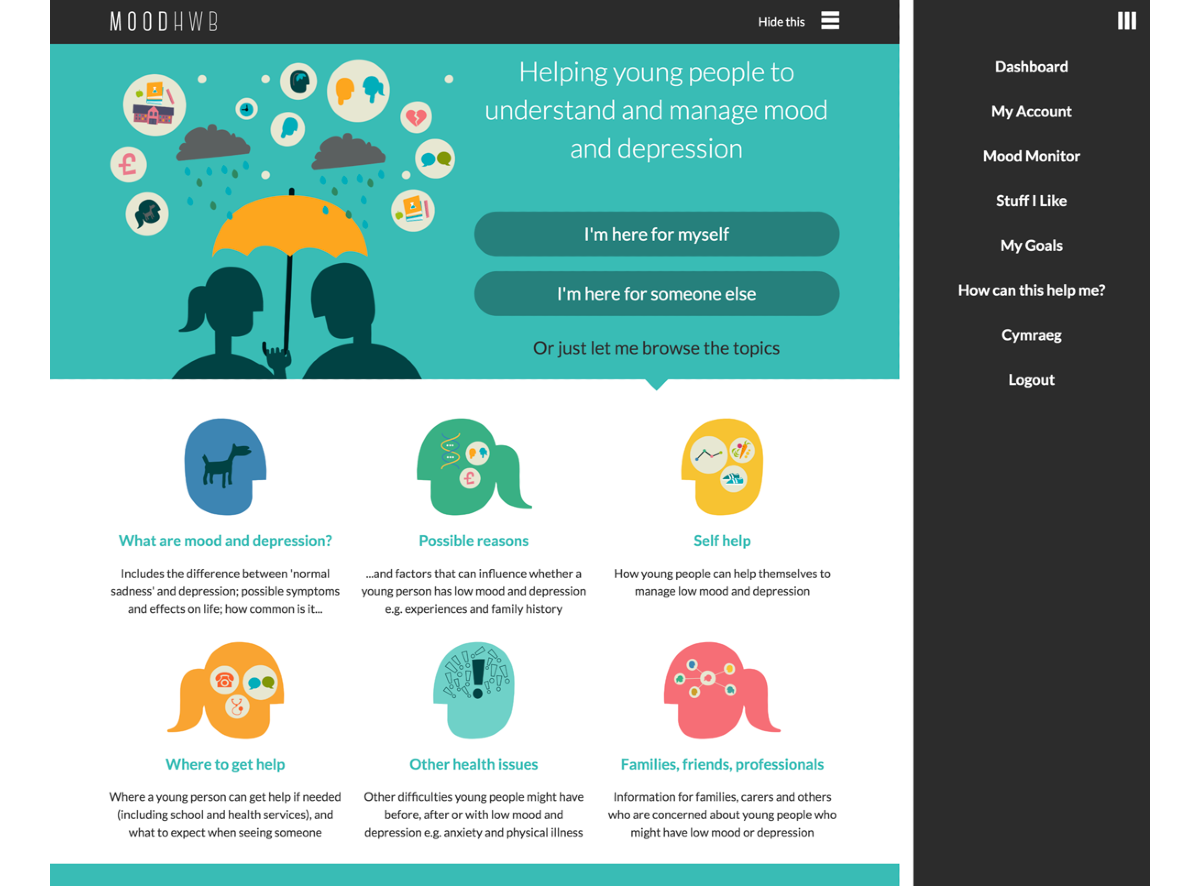
MoodHwb welcome screen (main image/left) and open menu (right).

**Figure 2:**
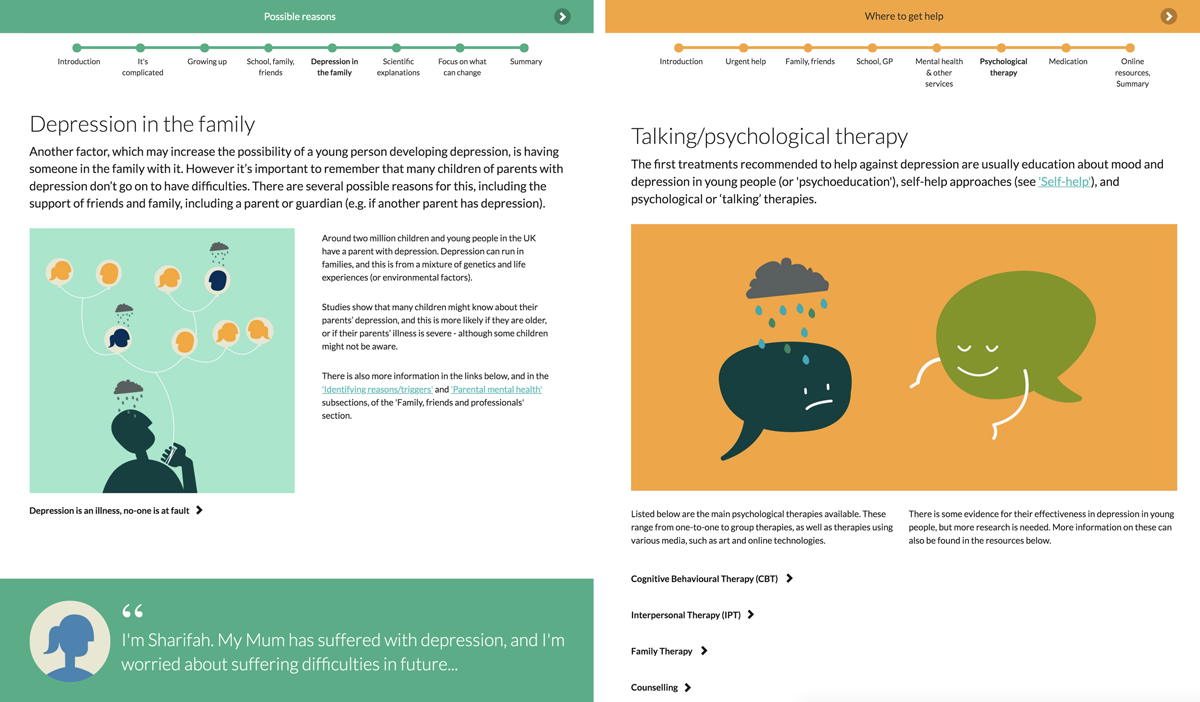
Examples of subsections from the “Possible reasons (for low mood)” and “Where to get help” sections.

The program includes sections on mood and depression, possible reasons for low mood and depression, self-management (including planning, problem solving, and lifestyle approaches), finding help, and other issues commonly experienced alongside depression (eg, anxiety; Figures 1 and 2). There is also a section for families, carers, friends, and professionals, in part to promote social support.

**Figure 3:**
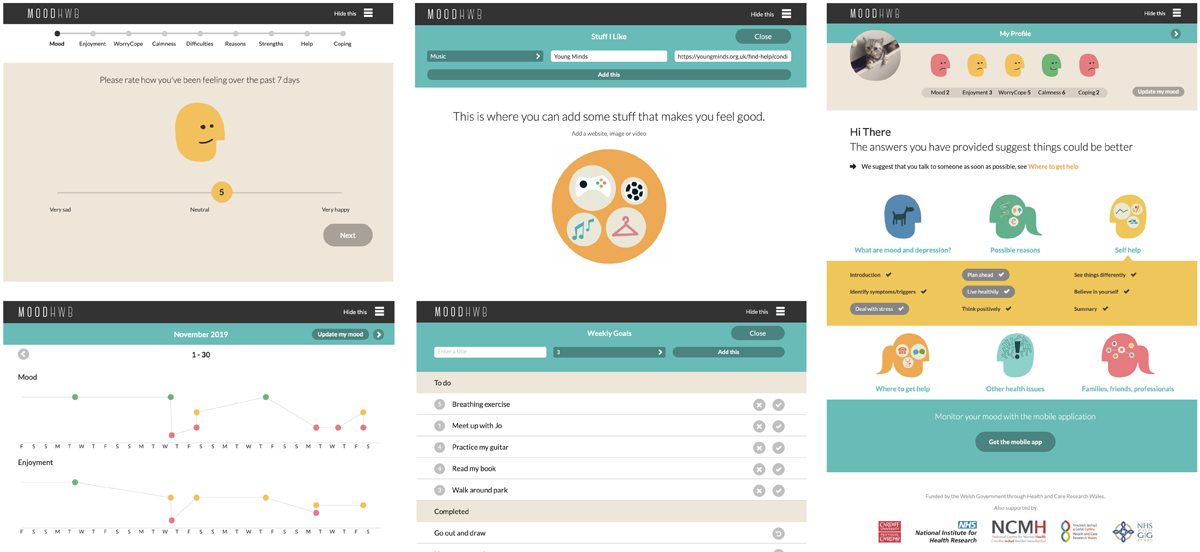
Interactive elements: mood monitor and diary (left); “Stuff I like” (top center); goal setting (bottom center); dashboard/profile section (right).

On the welcome screen, there are separate user pathways for the young person (“I’m here for myself”) and for the parent, carer or another person concerned about a young person (“I’m here for someone else;” Figure 1). The user is then asked questions (eg, regarding their mood and anxiety), and the answers (1) are stored in the “My profile” section, and (2) help to signpost the relevant subsections on the subsequent dashboard screen. Along with this “Mood monitor,” there are other interactive elements: a goal setting element (“My goals”) and a section to save links to helpful resources (“Stuff I like;” Figure 3).

MoodHwb is available in English and Welsh (*hwb* is the Welsh translation for *hub*, and can also mean a *lift* or *boost*—HwbHwyliau is the Welsh name for the program). The program and the design and development process are described further by Bevan Jones et al [21,22].

### Study Design

A mixed methods (quantitative and qualitative) approach was taken to assess the feasibility, acceptability, and potential effectiveness of the program. Data were collected from the following: (1) semistructured interviews with young people, parents and carers who used the intervention, (2) a focus group with professionals, (3) Web usage data, and (4) pre intervention and post intervention questionnaires.

### Ethical and Health Board Approval

The Dyfed-Powys (Wales, the United Kingdom) and Cardiff University School of Medicine Research Ethics Committees gave a favorable opinion for the research project. Research and development approval was granted by Cwm Taf, Cardiff and Vale, Abertawe Bro Morgannwg, Aneurin Bevan, Hywel Dda, and Powys University Health Boards (UHBs), 6 of the 7 UHBs in Wales. The authors of the questionnaires were contacted where approval was required.

### Participants

We planned to approach around 40 young people, as this sample size was considered sufficient to allow assessment of acceptability and feasibility in a relatively broad sample of the target group. Young people were recruited from specialist Child and Adolescent Mental Health Services (CAMHS), primary mental health teams, school counselors and nurses, and from the Cardiff Early Prediction of Adolescent Depression (EPAD) study [23]. Individuals (recruited from CAMHS and EPAD) who participated in the design and development phase of MoodHwb were also invited to participate.

#### Eligibility Criteria

Young people had to be at least 13 years of age, and to have either (1) a current or past history of depression or (2) be at elevated risk of depression due to a family history of recurrent depression in a parent. The parents and carers of these young people were also invited to take part. Participants were not eligible if they were unable to understand the intervention or questions/discussions (eg, because of insufficient understanding of English).

Professionals from health, education, social, and youth services and charities were approached if they worked with young people with mental health difficulties.

#### Recruitment and Consent

Eligible young people were identified by their CAMHS and primary mental health practitioners and school counselors and nurses, and provided with letters and information sheets. Individuals from the EPAD study (who had consented to being contacted about other research) were sent a letter and information sheet inviting them to participate. Interested young people and their parents or carers sent a completed reply card to the study team, who then telephoned the young person and parent or carer to discuss the project further. Participants were given the choice of meeting the study team to discuss the project and complete the consent forms in person, or to do this via post. Signed consent was obtained from young people above the age of 16 years, and signed parent and guardian consent and child assent from those under 16 years. Professionals from the appropriate services were identified and sent a letter and information sheet, inviting them to participate, with a reply card as above.

### Procedures

Consenting participants were provided a link to the program via email, and they created their own passwords. Parents and carers could create separate accounts to their children. Participants were informed that they could use the program as they wished (MoodHwb includes a section and animation to introduce the program to the user). Young people continued to attend sessions with their practitioner or counselor, if they were seeing someone. They were given access for a minimum of 2 months to allow them sufficient time to work through the program. Professionals were provided with a link to the program 2 weeks before their focus group.

#### Interviews and Focus Group

##### Semistructured Interviews With Young People, Parents, and Carers

Young people were asked whether they would like to be interviewed alone or with a parent or carer. The parent or carer was also asked whether they would like to be interviewed separately. Interviews were held either at Cardiff University or a location convenient for the participant (eg, home and school) and lasted up to 90 min. All interviews were completed by RBJ. The interviews were informal, and interviewees had access to MoodHwb on a mobile device.

##### Sampling Frame

Adolescents were selected for interview based on their usage of MoodHwb (ie, frequent or occasional), age, gender, service or charity involvement, and primary language (English, Welsh). The range of characteristics and number of interviews completed ensured a broad spread of viewpoints.

##### Focus Group With Professionals

The group session was held at Cardiff University and was facilitated by RBJ and a colleague. The session lasted approximately 120 min. The program was projected on a screen during the group session. We aimed for a balance of professionals in terms of services and charities, gender, and primary language (English, Welsh).

The interviews and focus group were all audio recorded digitally and transcribed; participants could also write or draw their thoughts.

#### Web Usage Data

Individual user interactions with the website were captured from bespoke analytics developed by the digital media company.

#### Pre Questionnaires and Post Questionnaires

Adolescent participants and their parents or carers were asked to complete questionnaires before and after having access to MoodHwb (after approximately 2 months). As with the consent forms, participants were given the choice of completing the questionnaires in person or via post (with phone or email support if required). Parents and carers completed questions about their child and about themselves.

### Measures

The main outcomes of the evaluation related to the feasibility and acceptability of the digital program and of the evaluation process. Secondary outcomes included the potential effect on mood, knowledge, attitudes, and behavior after using the program.

#### Feasibility and Acceptability of the Program

The feasibility of the program was assessed in part through information on usage. This consisted of (1) Web data, including the number of visits to each section, language used on each visit (English/Welsh), and user pathway (for self or another person); and (2) self-reported questionnaire data, including the frequency of use, sections looked at the most often, language option chosen (English, Welsh, both), average time spent on the site each time, and use with others (eg, parent, carer, friend, professional). Participants were also asked about technical issues they experienced in the interviews and focus group.

The acceptability of the program was assessed in the interviews, focus group, and feedback questionnaire. The interview topic guide covered (1) general views of the program, (2) specific strengths, (3) areas to develop further regarding the design and content of each section, and (4) integration into services and charities. The interviewing was iterative; where new themes emerged, they were incorporated into the subsequent interviews. The focus group discussion outline evolved from this guide.

#### Feasibility and Acceptability of the Evaluation Process

Assessment of the feasibility of the evaluation process included information related to the recruitment (number and range of participants) and retention of participants, as well as the completeness of data. Participants were also asked about the acceptability of the process in the interviews and via the pre intervention and post intervention questionnaires (eg, regarding their views on the number of questions).

#### Potential Impact

Standardized questionnaires were used to explore changes in depression literacy and stigma, help-seeking behavior, self-efficacy, behavioral activation, depression and anxiety symptoms, and general behavior (see Table 1 for the list of measures [24-32]).

**Table 1:** Content of standardized questionnaires (pre intervention and post intervention).

Outcome	Questionnaire	Rater
Depression literacy	Adolescent Depression Knowledge Questionnaire [24]	Child self-report; parent/carer self-report
Depression stigma	Depression Stigma Scale [25]	Child self-report; parent/carer self-report
Help-seeking behavior	General Help-Seeking Questionnaire [26]	Child self-report
Self-efficacy	Self-Efficacy Questionnaire for Depression in Adolescents [27]	Child self-report
Behavioral activation	Behavioral Activation for Depression Scale [28]	Child self-report
Depression symptoms (child)	Mood and Feelings Questionnaire [29]	Child self-report; parent/carer about child
Anxiety symptoms	Screen for Child Anxiety Related Emotional Disorders [30]	Child self-report; parent/carer about child
General behavior, strengths, and difficulties	Strengths and Difficulties Questionnaire [31]	Child self-report; parent/carer about child
Depression and anxiety symptoms (parent or carer)	Hospital Anxiety and Depression Scale [32]	Parent/carer self-report

### Data Analysis

#### Qualitative Analysis

The interview and focus group transcripts were analyzed using a thematic analysis approach [33]. To ensure reliability of coding, all transcripts were coded by RBJ, and 40% of transcripts were double coded independently by SSA. Coding a proportion of interviews (from around 10% of transcripts) is a standard approach to improve coding reliability [34,35], and we agreed this higher proportion in advance as it was likely to increase the rigor of coding. We did not measure concordance in a quantitative way (eg, using kappa statistic), as this is controversial in qualitative research [36]. Agreement on concepts and coding was also sought with other authors (SS, AT, and BM). Initial ideas on the coding framework were discussed among the team; the draft framework was applied to some of the data and refined as coding proceeded. Codes were applied to broad themes, which were then broken down further into subcodes. Transcripts were closely examined to identify the key themes and associated subthemes. Thematic analysis was supported by the computer-assisted NVivo qualitative data analysis software (version 10, QSR International Pty Ltd).

#### Quantitative Analysis

Quantitative data from the questionnaires and Web usage were analyzed and presented descriptively with summary statistics (means, percentages). Paired sample *t* tests were used to explore changes in outcome scores between the pre intervention and post intervention questionnaires (the prescores were subtracted from the postscores). Findings from the Shapiro-Wilk test did not show statistical evidence to suggest a deviation from normality for any of the reported outcomes. Mean differences and confidence intervals were presented for these analyses. When generating sum scores for the individual pre questionnaires and post questionnaires, missing values were replaced with the mean value for that individual, provided there was less than 10% of missing data. All analyses were conducted using Stata statistical software (version 14, StataCorp LP).

## Results

### Participants

**Figure 4:**
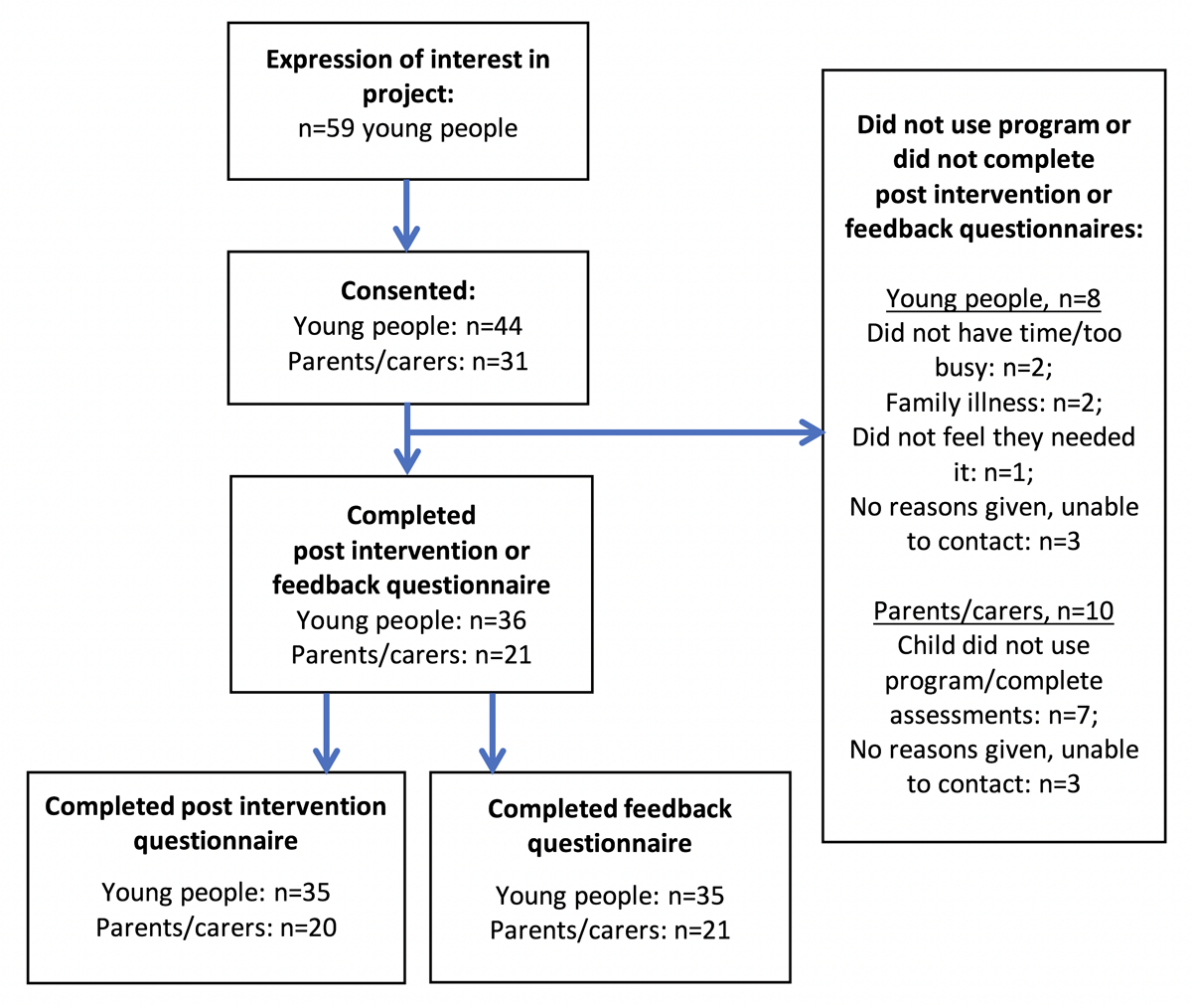
Flowchart of participants in the quantitative evaluation phase.

Figure 4 shows the flow of participants in the quantitative evaluation phase. In total, 59 young people expressed an interest in participating in the evaluation phase, and 44 consented to participate (75%). Of those who consented to participate, 36 young people (82%) completed either the post intervention or feedback questionnaire (ie, 2 of the young people completed only one questionnaire). In all, 31 parents or carers provided consent to participate, of whom 21 (68%) completed either the post intervention or feedback questionnaire (ie, all but one of the parents or carers completed both questionnaires).

The characteristics of participating young people are presented in Table 2. The ratio of females to males was approximately 4:1. The majority (29/31, 94%) of the parents and carers who participated were mothers. In all, 45% (14/31) of the parents and carers were receiving treatment for depression, and 72% (21/29) had been treated for depression during their lifetime. Of those who participated in the previous development phase, 17 young people and 6 parents or carers consented to participate in the evaluation (ie, 39% of young people and 19% of parents and carers who consented to participate). The levels of missing data are shown in the footnotes of the tables below, where relevant.

**Table 2:** Characteristics of young people participating in the study at baseline (N=43).

Characteristics^a^		Value
**Recruitment source, n (%)**		
	School counselor or nurse	8 (19)
	Primary mental health team	6 (14)
	Specialist CAMHS^b^	10 (23)
	EPAD^c^ study	13 (30)
	Volunteer	6 (14)
**Age (years)**		
	Mean (SD)	16.3 (2.36)
	Median	16
	Range	13-23
**Gender, n (%)**		
	Female	34 (79)
	Male	9 (21)
**Currently getting help, n (%)**		
	School counselor or nurse	14 (33)
	General practitioner	9 (21)
	Youth worker	3 (7)
	Social worker	1 (2)
	Mental health worker	14 (33)
	Other	3 (7)
Baseline depressive symptoms (MFQ^d^), mean (SD)		34.4 (15.46)
Currently attending sessions for psychological therapy for low mood/depression^e^, n (%)		21 (62)
Currently prescribed medication for depression^e^, n (%)		13 (38)
Past treatment for depression^e^, n (%)		9 (22)

^a^Data available for 43 out of 44 participants, as one participant did not complete the pre intervention questionnaire.

^b^CAMHS: Child and Adolescent Mental Health Services.

^c^EPAD: Early Prediction of Adolescent Depression.

^d^MFQ: Mood and Feelings Questionnaire.

^e^Number with missing data was 1 for MFQ, 9 for psychological therapy, 9 for medication, and 2 for past treatment.

Descriptive information about interview and focus group participants is provided in Table 3. A total of 19 young people were interviewed; the ratio of females to males was approximately 3:1. Approximately two-thirds of the young people were interviewed along with their parents or carers (11 mothers, 1 father). In all, 9 of those interviewed spoke Welsh fluently. A total of 13 professionals from a range of disciplines participated in the focus group.

**Table 3:** Characteristics of young people interviewed and professionals in the focus group.

Characteristics				Value	
	**Interviews for young people (n=19)**				
		**Recruitment source, n (%)**			
			Mental health service or school counselor/nurse		13 (68)
			EPAD^a^ group (parent with depression)		6 (32)
		**Age (years)**			
			Mean (SD)		16.5 (1.78)
			Median		16
			Range		14-19
		**Gender, n (%)**			
			Female		14 (74)
			Male		5 (26)
		**Ethnicity, n (%)**			
			White		18 (95)
			Other		1 (5)
		Seen alone, n (%)			7 (37)
		Seen with parent or carer, n (%)			12 (63)
		**Interview location, n (%)**			
			Home address		12 (63)
			Cardiff University		6 (32)
			School		1 (5)
	**Focus group for professionals (n=13)**				
		**Profession, n (%)**			
			Psychiatrist		2 (15)
			Mental health nurse (secondary care)		1 (8)
			Primary mental health worker		2 (15)
			Social worker		1 (8)
			Educational psychologist		2 (15)
			School nurse		1 (8)
			School counselor		1 (8)
			Teacher		1 (8)
			Youth worker and community wellbeing officer		1 (8)
			Charity worker (emotional well-being and mental health manager)		1 (8)
		**Gender, n (%)**			
			Female		10 (77)
			Male		3 (23)
		**Ethnicity, n (%)**			
			White		13 (100)

^a^EPAD: Early Prediction of Adolescent Depression.

### Use of the Program

#### Web Usage Data

Analytics from the digital company revealed that the most common section accessed by young people, parents and carers was “What are mood and depression?” (27% of total use). The other sections were accessed approximately half as often (range 12%-17%). Overall, 16% of usage was in Welsh and 84% was in English. Most participants used the program for themselves (78% of use) and 22% for another person.

#### Questionnaire Data

The questionnaire findings on usage are presented in Table 4. In total, 21% (7/34) of young people used it once or twice a week, 44% (15/34) used it once or twice a month, and 26% (9/34) used it once or twice overall. Parents and carers used it less frequently, with most (11/20, 55%) using it only once or twice. A total of 30% (6/20) of parents and carers used it once or twice a month, and 10% (2/20) used it once or twice a week. Most young people (19/35, 54%), parents and carers (12/20, 60%) used it for half an hour at a time and most young people used it alone (29/33, 88%). Parents and carers were more likely to use it with others (7/19, 37%).

Both groups reported looking the most at the “Self help,” “What are mood and depression?,” and “Possible reasons” sections. Young people also reported they looked at the “Mood monitor,” and parents and carers looked at the “Families, carers, friends, professionals” section.

#### Interviews and Focus Group

There were some difficulties related to the compatibility of the program with certain devices and operating systems, especially NHS computers. Some experienced difficulties with internet access at home or on mobile devices.

**Table 4:** Use of program (questionnaire data).

Use of program		Young people^a^ (n=35), n (%)	Parents or carers^b^ (n=20), n (%)
**Frequency of use**			
	Nearly every day	1 (3)	0 (0)
	3-4 times a week	1 (3)	0 (0)
	Once or twice a week	7 (21)	2 (10)
	Once or twice a month	15 (44)	6 (30)
	Once or twice overall	9 (26)	11 (55)
	Not used	1 (3)	1 (5)
**For how long the program was used each time?^c^**			
	Several hours	1 (3)	0 (0)
	About an hour	10 (29)	4 (20)
	About half an hour	19 (54)	12 (60)
	Few minutes	7 (20)	4 (20)
	Not used	1 (3)	2 (10)
**Sections looked at the most^c,d^**			
	My profile	16 (47)	3 (16)
	What are mood and depression?	17 (50)	13 (68)
	Possible reasons	15 (44)	10 (53)
	Self help	23 (68)	10 (53)
	Where to get help	8 (24)	6 (32)
	Other health issues	9 (26)	3 (16)
	Families, carers, friends, professionals	7 (21)	8 (42)
	Mood monitor	22 (65)	4 (21)
	Stuff I like	9 (26)	1 (5)
	My goals	7 (21)	1 (5)
	The app	10 (29)	3 (16)
**Language**			
	English	27 (79)	18 (95)
	Welsh	2 (6)	1 (5)
	Both	5 (15)	0 (0)
**Use with others^c^**			
	Used with others	4 (12)	7 (37)
	Parent or carer	3 (9)	N/A
	Friend	1 (3)	0 (0)
	Professional	0 (0)	0 (0)
	Young person	N/A^e^	7 (37)
	Partner	N/A	0 (0)
	Other	0 (0)	0 (0)

^a^Data available for 35 out of 36 young people. The number with missing data was 1 for frequency of use, 1 for sections looked at the most, 1 for language, and 2 for use with others.

^b^Data available for 20 out of 21 parents/carers. The number with missing data was 1 for sections looked at the most, 1 for language, and 1 for use with others.

^c^Participants could select more than one response option.

^d^Participants were asked “Which sections did you look at the most? (please select all that apply).”

^e^N/A: not applicable.

### Views/Acceptability of the Program (Results of Interviews and Focus Group)

The overarching themes in the interviews and later in the focus group were as follows: (1) design features, (2) sections and content, and (3) integration and context. These themes were influenced by the primary issues the research team wished to explore, to help refine the content and design of MoodHwb. The overall feedback from participants is presented later, with verbatim examples listed in Table 5. Specific suggestions on changes to the program are described in Multimedia Appendix 1. Many of those who had participated in the development phase commented that they were pleased to see that suggestions made in the codevelopment group sessions had been adopted in the prototype.

#### Key Theme 1: Design Features

##### Overall Design, Navigation, and Ease of Use

In general, all interview and focus group participants made favorable comments about MoodHwb and stated that it seemed helpful for young people, families and carers. Some noted they were surprised by the high quality, and that they had reservations about it previously because it might be “academic,” “dry,” or “overwhelming.” Most found the overall design attractive, and more interesting and appropriate than designs for existing resources (quote 1).

Overall, participants felt that the program was clearly and consistently structured, easily navigated and user-friendly. They noted that the design and layout made it easy to identify what was relevant. Participants found the color coding, progress bar (see Figure 2), and quizzes particularly engaging (quote 2), but suggested that some elements could be better signposted.

##### Interactive Elements and Personalization

Figure 3 presents screenshots from the interactive elements of the program.

###### Initial Questions and Mood Monitoring

Nearly all praised the mix of rating scales (and corresponding face icons) and multiple-choice questions at the start of the program (which could also be answered subsequently to monitor mood), particularly for their functionality, ease of use, and “fun” element. Some noted that this was their favorite aspect (quote 3). Several professionals approved the message for the user to seek help, if they indicated they had thoughts of self-harm.

####### “Stuff I Like”

Some young people had used this to add songs and images, and felt that it helped to personalize the program. However, others had not done so, because they already bookmarked sites they found interesting.

####### Goal Setting

Most found “My goals” to be helpful and motivating. They liked how it was possible to add the number of activities, and to note when they were completed (quote 4).

A few noted that their use of these interactive elements phased out over time, and that reminders and rewards might increase engagement. They also noted these components could be explained and personalized further.

####### Dashboard and Profile Section

“My profile” was described as helpful, particularly to group together strengths and difficulties and save links to resources. Professionals noted that this section helped users to reflect on and add perspective to their situation (quote 5).

##### Illustrations and Animations

Young people, parents and carers described the illustrations and animations as “friendly” and “sophisticated” without being “patronizing.” The characters were designed to appeal to a diverse audience, with abstract minimal features. All young people stated they preferred the illustrative approach to a more photographic one (quote 6).

In summary, participants, in particular young people, gave favorable feedback on the design features, including the overall presentation, structure, and interactive and graphic elements. They also suggested the areas to develop further, including the navigation, personalized components, interactive and audiovisual elements, reminders, and rewards, and “native app.”

#### Key Theme 2: Sections and Content

##### Language, Tone, and Amount of Information

On the whole, participants agreed that the language used was accessible, simple, and jargon-free, but “not too dumbed down.” Many approved of the general tone, stating it was “sensitive” and “affirming” (quote 7).

In general, participants were pleased with the content, and found it comprehensive. They liked the flow of information, and how text was broken up (eg, by illustrations), “bite size,” and “short and snappy.”

Professionals praised the “extensive,” “authoritative,” and “reliable” information. There were mixed views regarding the amount of text—some felt there was too much, others that it was appropriate (quote 8).

##### Personal Stories

All participants made favorable comments about the personal stories, and how they were from a range of perspectives (quote 9). Young people, parents, carers, and a small number of the professionals suggested adding stories from “celebrities,” and including photos, animations, videos, or comic strips.

**Table 5:** Themes, subthemes, and quotes from interviews and focus group.

Themes and subthemes		Quote No.	Verbatim examples
**Key theme 1: Design features**			
	Overall design, navigation, and ease of use	1	“I was really pleasantly surprised with it…It was great and I can definitely see people using it and wanting to use it. I was very impressed with it. Yeah, I wish I could criticise a bit more, but I can’t.” (19-year-old female)
		2	“[It’s] accessible to a whole range of people…[They] very quickly understand where they needed to go.” (Mother of a 15-year-old female)
	Interactive elements and personalization	3	“It was good to be able to see how you’ve been each day…It’s just helpful to track, that’s not something I would usually do.” (19-year-old female)
		4	“That was really helpful for myself because I've been trying to get out of the house and do more…That was one of my favourite parts…It's quite motivating.” (16-year-old female)
		5	“I like the fact that there was space for the young person to add their own contacts, so they then had ownership of it; it’s given them a bit of responsibility.” (Primary mental health worker: female)
	Illustrations and animations	6	“I think the illustrations…are very good…it’s not a chore to go and look at the website…It’s not that it’s childish, but…it’s less serious, I think it’s easier to use.” (16-year-old female)
**Key theme 2: Sections, content**			
	Language, tone, and amount of information	7	“It's not too complicated, so teenagers can understand it and relate to it…There's no ridiculously big words and [it’s not too] scientific. It's a good style of writing to keep teenagers reading.” (17-year-old female)
		8	“We’ve got to be careful not to lower it too much because they’ve actually got onto this website because they’re needing information.” (Educational psychologist: female)
	Personal stories	9	“I do like the personal stories because then you feel like you're not the only person that’s going through a hard time. You can maybe relate as well.” (16-year-old male)
	Sections	10	“I’ve used it a lot since being diagnosed and it helps me to understand what depression is and some of the reasons.” (15-year-old female)
		11	“It's not something, especially if you're in a really low mood, you particularly want to think about…It's just talking about things that you try and avoid basically.” (16-year-old female)
		12	“What’s really good is it’s relevant…it can be due to all sorts of different things so… for a young person coming to realize actually it’s not just about me and now and just one thing.” (Psychiatrist: female)
		13	“The sections I went to is perfect, it’s exactly how I imagined it to be, like self help, I particularly like that section.” (16-year-old male)
		14	“My only concern with that is you run the risk then of the people who are a bit paranoid is self‑diagnosis…and become panicky and anxious.” (Mother of a 17-year-old female)
		15	“What I really liked about it is that the fact that it’s talking more about the family… It’s recognizing that the children’s mental health difficulties don’t come in isolation.” (Psychiatrist: female)
		16	“From a friend’s perspective, it’s quite difficult to start a conversation about depression.” (14-year-old-male)
**Key theme 3: Integration and context**			
	Targeted versus universal	17	“Whoever looks at it it’s gonna be beneficial. There’s a lot of information on there that even if you haven’t got a mental health problem or maybe you know someone or even if you’re just curious.” (19-year-old female)
	Use with families, carers, and friends	18	“In mine and my friends’ experiences, the hardest people to talk to would be parents, so I like this because it’s a way that you can use the account separately, but learn the same things.” (15-year-old female)
	Integration with services	19	“It’s there to back it up at home...So between the sessions you’ve got this at home to use…. A safety net or when I stop CAMHS sessions then it’s just there when I need it.” (14-year-old female)
		20	“A big part of our work is psychoeducation and so it would be great to know that there’s a reliable, moderated place that you can send them, because at the moment we tend to use sites that are adult-based and we’re having to cut and paste.” (Psychiatrist: female)

##### Sections

Figure 1 shows how the sections are displayed on the welcome screen, and Figure 2 presents screenshots of two of the subsections.

###### What Are Mood and Depression?

Participants approved of the information on the difference between sadness and depression, how to identify difficulties and associated metaphors (quote 10). Some noted that those with depression might be reluctant to engage with the subject matter (quote 11).

###### Possible Reasons for Low Mood and Depression

Professionals were particularly pleased with this “exploratory” section (Figure 2; quote 12).

###### Help Sections

Several stated that the self help section was “motivational” and their favorite section (quote 13), and some asked for more self help approaches in specific situations. Professionals approved of the description of services and professionals (Figure 2), and of signposting to resources.

###### Other Health Issues

Most noted that the information was important and comprehensive, particularly on anxiety, eating and weight issues, and physical health. However, a few asked for more specific information (eg, on panic), while others were concerned that some users might worry unnecessarily (quote 14).

###### Families, Carers, Friends, and Professionals

Parents, carers, and professionals praised the “holistic” and “systemic” approach and highlighted the parental mental health subsection (quote 15). Some suggested adding more information on how to talk to a family member or friend (quote 16).

Generally, participants approved of most aspects of the content, including the language, tone, personal stories, and the individual sections of the program. Areas to improve upon related to the specific information and amount of text in general.

#### Key Theme 3: Integration and Context

##### Targeted Versus Universal

Most participants (and all professionals) thought that MoodHwb would be engaging and helpful for anyone who was interested in learning more about mood and depression (quote 17). Some of those who had not experienced depressive difficulties (from the EPAD study) did not think the program was as relevant to them, while some of those who had completed CAMHS therapy sessions already knew much of the information included in the program. On the whole, participants concluded that MoodHwb was especially appropriate for those starting to experience difficulties. Some stated that the program could help counteract stigma and making it freely available would help with this.

##### Use With Families, Carers, and Friends

Most young people had separate user accounts to their parents and carers; some suggested adding the option to monitor someone else’s mood and share information. Participants felt that the program could help with communication between young people and their family, carers and friends (quote 18).

##### Integration With Services

Many felt that MoodHwb could be used in schools, particularly personal, social and health education lessons – and suggested including a “teacher area” within the program. The school-based professionals stated that it could address the “big gap” in resources and training for teachers. However, some young people noted that associating it with schools might make it less appealing.

Some noted that the use of MoodHwb could complement existing services, for example, by allowing young people to reflect on their entries with counselors, General Practitioners, and primary mental health and specialist CAMHS practitioners (quote 19).

All health care professionals felt that the intervention could be used at their workplace—especially at the school and primary care level. Some noted that it could be helpful for those no longer in services, waiting for an appointment or who did not meet the criteria for mental health services. An educational psychologist noted that the digital program would be relevant for children in care, especially as “they’re moving around a lot” (quote 20).

Many professionals highlighted the challenge of keeping the program up to date. One psychiatrist advised that many professionals were not IT-literate and might be reluctant to recommend it.

In summary, most participants felt that all young people, families and carers should have access to MoodHwb, and it would be especially helpful as an early intervention in a range of settings for those who were starting to experience difficulties.

##### Views and Acceptability of the Program (Results of Feedback Questionnaire)

Overall, young people reported that the program was helpful, especially with finding ways to help themselves (Multimedia Appendix 2). When asked to select all the sections they found particularly helpful, most young people identified “Self help” (26/35, 74%), followed by “Mood monitor” (21/35, 60%), “What are mood and depression?” (19/35, 54%), and “Possible reasons” (19/35, 54%). The sections identified as the least helpful were “Stuff I like” (13/35, 37%), “Families, carers, friends, professionals” (7/35, 20%), and “My goals” (7/35, 20%), although interview feedback about these sections was more favorable. In total, 51% (17/33) downloaded the accompanying app and 14% (5/35) felt this was one of the most useful components.

Findings were similar for parents and carers; they felt that the young person found it helpful overall and selected “Self help” (9/21, 43%) and the “Mood monitor” (8/21, 38%) as the most useful for the young person. Overall parents and carers found the program helpful for themselves (Multimedia Appendix 2). The sections identified as most helpful were “Possible reasons” (13/21, 62%), “What are mood and depression?” (11/21, 52%), “Where to get help” (9/21, 43%), “Families, carers, friends, professionals” (8/21, 38%), and “Self help” (8/21, 38%).

Overall, young people, parents, and carers felt that the amount of information included in each section was adequate (Multimedia Appendix 2). Both groups rated the ease of use and clarity highly, and approved of the design elements, although parents and carers were slightly less positive than young people (Multimedia Appendix 2).

### Acceptability of Interviews and Questionnaires

During the interviews, young people stated they were able to discuss the program openly and appreciated that they could choose the location, and whether they were seen with their parents or carers. They found it helpful to be able to navigate through the program as it was discussed.

Data on acceptability of questionnaires were available for 43 young people and 30 parents or carers at baseline, and 34 young people and 19 parents or carers at follow-up. Most felt the number of questions was “about right.” Young people at follow-up were more likely to rate the questionnaires as having a “few too many questions” than at baseline, possibly due to the additional burden of the feedback questions.

### Potential Effect of the Program (Comparison of Pre intervention and Post intervention Questionnaires)

Table 6 summarizes the results from the pre intervention and post intervention standardized questionnaires. It is important to note that these findings are considered exploratory given the small sample size and lack of power.

#### Young People

Levels of depression literacy were higher after using the program (difference in means 1.7, 95% CI 0.8 to 2.6). Although the differences were small, several other scores also improved, particularly regarding self-efficacy and depressive symptoms.

#### Parents and carers

Parents’ and carers’ own depression literacy improved after using the program (difference in means 1.3, 95% CI 0.4 to 2.2). Parent-rated scores of their children’s mood and behavior were similar or slightly worse after using the intervention. Overall, parent-rated scores of their child’s depression, anxiety, and behavior were lower than children’s self-rated scores.

**Table 6:** Comparison of pre intervention and post intervention questionnaires.

Outcomes (Questionnaires)		Pre intervention, mean (SD)			Post intervention, mean (SD)		Difference in means (95% CI)		*t* test (*df*)		*P* value	
**Young people^a^ (n=35)**												
	Depression literacy (ADKQ^b^)		9.1 (1.95)			10.8 (2.24)		1.7 (0.8 to 2.6)		3.82 (29)		<.001
	Depression stigma (DSS^c^)		30.3 (6.39)			29.4 (5.89)		–0.7 (–3.2 to 1.7)		-0.62 (32)		.54
	Self-efficacy (SEQ-DA^d^)		30.3 (6.67)			32.0 (8.85)		1.7 (–1.0 to 4.5)		1.28 (33)		.21
	Help-seeking (GHSQ^e^)		69.2 (15.2)			71.8 (14.04)		2.6 (–3.1 to 8.4)		0.95 (28)		.35
	Depression (MFQ^f^)		36.5 (15.13)			33.9 (16.48)		–2.6 (–7.2 to 2.0)		–1.16 (33)		.26
	Behavioral activation (BADS^g^)		71.9 (24.07)			72.7 (29.13)		0.8 (–8.5 to 10.2)		0.18 (34)		.86
	Anxiety (SCARED^h^)		42.2 (18.04)			40.6 (18.46)		–1.6 (–5.4 to 2.2)		–0.87 (34)		.39
	Behavior (SDQ^i^)		17.4 (6.33)			17.4 (6.63)		0.06 (−1.5 to 1.7)		0.08 (33)		.94
**Parent/carer rated about their child^j^ (n=20)**												
	Depression (MFQ)		21.8 (15.47)	24.2 (14.3)				2.4 (–2.1 to 7.0)		1.13 (16)		.27
	Anxiety (SCARED)		22.3 (15.41)	23.5 (12.32)				1.13 (–2.9 to 5.1)		0.60 (17)		.56
	Behavior (SDQ)		12.8 (7.24)	13.3 (5.14)				0.5 (–1.4 to 2.4)		0.55 (19)		.59
**Parent rated about themselves^j^ (N=20)**												
	Depression literacy (ADKQ)		9.6 (2.11)	11.0 (1.74)				1.3 (0.4 to 2.2)		3.07 (18)		.006
	Depression stigma (DSS)		30.4 (8.24)	30.2 (7.71)				–0.2 (–1.7 to 1.4)		–0.23 (17)		.82
	Depression (HADS^k^)		14.4 (8.61)	15.6 (8.79)				1.2 (–1.1 to 3.5)		1.13 (18)		.27

^a^Data available for 35 out of 36 young people. The number with missing data was 5 for the ADKQ, 2 for the DSS, 1 for the SEQ-DA, 6 for the GHSQ, 1 for the MFQ, 0 for the BADS, 0 for the SCARED, and 1 for the SDQ.

^b^ADKQ: Adolescent Depression Knowledge Questionnaire.

^c^DSS: Depression Stigma Scale.

^d^SEQ-DA: Self-Efficacy Questionnaire for Depression in Adolescents.

^e^GHSQ: General Help-Seeking Questionnaire.

^f^MFQ: Mood and Feelings Questionnaire.

^g^BADS: Behavioral Activation for Depression Scale.

^h^SCARED: Screen for Child Anxiety Related Emotional Disorders.

^i^SDQ: Strengths and Difficulties Questionnaire.

^j^Data available for 20 out of 21 parents/carers. The number with missing data was 3 for the MFQ, 2 for the SCARED, 0 for the SDQ, 1 for the ADKQ, 2 for the DSS, and 1 for the HADS.

^k^HADS: Hospital Anxiety and Depression Scale.

## Discussion

We aimed to conduct an early-stage evaluation of a novel digital psychosocial intervention for adolescent depression and to assess the feasibility and acceptability of the assessment process that will be used in a future trial.

### The Digital Intervention: MoodHwb

Most participants noted that the program was engaging, clear, easy to navigate, and well structured, and praised the graphic and animated approach. The content was described as comprehensive, motivational, holistic, and accessible to young people—and also relevant to families, carers and professionals in a range of services. Young people found it easier to use overall and were better able to understand the design elements, although parents, carers and professionals found it acceptable.

Nearly all who completed the follow-up used the program, with most young people using it alone and for at least half an hour at a time over the course of the study (minimum of 2 months), which is favorable in terms of adherence in eHealth [12]. Participants did not use it frequently; however, they found it helpful overall, and its use might vary according to the context and needs of the user (eg, the stage and severity of their difficulties) [37].

There were reports of improved depression literacy, self-efficacy (especially regarding self help strategies and knowing where to get help), and depressive symptoms—giving preliminary support for the intervention program theory [22]. There was no clear evidence of potential negative effects from using the program.

These results were consistent with a recent systematic review of psychoeducational interventions in adolescent depression [11], which found that these programs can be acceptable and feasible and can influence a range of outcomes. Overall, the development and initial evaluation of MoodHwb fills a knowledge gap identified in the review (particularly of digital programs) and meets the need for good health information and evidence-based psychosocial interventions [5].

MoodHwb might be particularly helpful when a young person starts to experience difficulties and first presents to services—for example to school counseling or nursing, primary care/mental health, youth, or social services. However, given that the majority of young people with depression do not access services [38], digital health interventions such as this could help improve reach and access to therapies. The program could also complement other approaches, for example, in the management of severe or chronic difficulties. It fits with the guided self help approach (as it can be used either independently or with another person) and the increasing interest in personalized medicine [39]. 

This evaluation will be used to inform the refinement of MoodHwb, with the aim of improving the user engagement with it. The suggestions for improvement made by participants were discussed with the digital media company and research team. The issues were prioritized according to the level of importance given to them by the participants, and whether they would improve the acceptability, feasibility, and ease of use of the program. Other considerations were the program aims, underlying theory and evidence, technical difficulty, and time, resources, and funds required to make the changes.

The main revisions considered included improving the navigation and audiovisual/interactive elements, making it more personalized, developing the app version of the program, and expanding the self help and anxiety sections (Multimedia Appendix 1). Ahead of a large trial, we would also need to ensure the program has the required technical specifications (eg, compatibility with devices and operating systems, adequate database capacity, and easy access to the app).

### The Evaluation Process

#### Strengths

One of the main strengths of the project relates to the rigorous mixed methodology, which followed guidance for complex interventions [20] for both the development and initial evaluation reported here. There was also a collaborative, person-based, and iterative approach [17] for the intervention development.

Another strength was the diverse range of participants from recruitment centers in urban and rural areas, including in areas with several ethnicities and areas of deprivation. Efforts were made to engage young people, families, and carers by offering to see them where convenient. There was also contact afterwards to promote retention and adherence (eg, email, text, phone). The recruitment target of 40 young people was met, and there was a good retention rate (82% of young people). The digital media team was available to address technical issues during the study.

#### Limitations

The research and digital media teams were involved throughout the project, and it is possible that their personal views could have influenced the development and evaluation process. Considering the number of centers visited, only a small number of potential participants were referred, which may have led to a biased sample. Several of those who initially showed interest did not go on to participate, and often no reason was given. This might reflect the difficulty in engaging young people in mental health research, or that the program might be more useful for certain subgroups or contexts. More females than males participated, although this might reflect in part that depression is more common in females in adolescence [1].

Participants might have reported more favorable responses because they were seen by, or in regular contact with, the researchers. Some had participated in the development phase, which may have influenced their opinion of the program (those who had been involved in its development generally gave favorable feedback on the prototype). Outcomes were assessed via self-report and response bias could be an issue, for example, where participants provide socially desirable answers. Young people might have been less likely to give candid responses in the presence of parents or carers, although they were given the option of being seen alone.

The results of the questionnaires must be interpreted with caution given the small sample size and limited power. It would have been interesting to explore how factors (eg, age, gender, severity/history of difficulties, family history, and involvement of parent and carer) might have influenced the results; however, the exploration of subgroups was not feasible given the sample size. It is also possible that some of those whose symptoms or behavior improved may have done so in any case over time, or due to other interventions or factors.

### Conclusions

This early mixed methods evaluation found that the co-designed prototype of MoodHwb and assessment process were feasible and acceptable. The findings will be used to refine the intervention and inform the design of a randomized controlled trial [20]. If proven to be effective, MoodHwb could ultimately be rolled out as an early intervention program in health, education, social, and youth services and charities to help young people, families, carers, friends, and professionals.

## Appendix

Multimedia Appendix 1 Table 1: Suggestions for the further development of the program; Table 2: Further quotes from the interviews and focus group.

Multimedia Appendix 2 Questionnaire feedback on program.

## Abbreviations

CAMHS: Child and Adolescent Mental Health Services
eHealth: electronic health
EPAD: Early Prediction of Adolescent Depression
UHBs: University Health Boards
