# Supplementary material for: A Digital Intervention for Adolescent Depression (MoodHwb): Mixed Methods Feasibility Evaluation
Source: JMIR Ment Health. 2020 Jul 17;7(7):e14536. doi: 10.2196/14536 (PMC7395255; doi:10.2196/14536)
Supplement: Multimedia Appendix 1 [file mental_v7i7e14536_app1.docx]

**Table 1: Suggestions for the further development of the program**

| **Suggestions by participants** (by young people and parents/carers unless stated otherwise) | | **Plans to address suggestions** |
| --- | --- | --- |
| **Specific design issues** | | |
| **Login screen** | Add a ‘remember me’ option (which might make it more likely for some to re-enter), although possible confidentiality issue | Add this option |
| **Welcome screen**  **(or home screen)** | Elements could be clearer on this screen:   - Introduce a link to ‘urgent help’ - Clearer reference to the Welsh language option (for example with a flag) - Add an explanation of ‘hide me’   There could be a link to the welcome/home screen throughout the package, and an option to return to the welcome screen instead of the dashboard.  The meaning of ‘MoodHwb’ could be more explicit, especially the translation of ‘hwb’.  There could be a brighter, lighter background colour for the welcome screen, such as a light orange, although most preferred keeping the teal  Suggestions by professionals:  Design a clearer ‘home’ button, and the menu could go at the bottom as well as the top of the screen.  A new idea was that there could be information on how the package was created.  A brighter colour to the welcome screen, but should also consult young people. | To explore how to optimise ‘signposting’ and ‘navigation’, and revise the design of the dashboard in line with the welcome screen.  Footnote to be introduced at the bottom of each screen to explain the name.  Background colour to remain teal  Page to be introduced on how the package was developed |
| **Menu icon** | Most parents/carers did not understand this icon, and this could possibly be clearer. | Explore how to make this clearer e.g. add term ‘menu’ |
| **Introductory animation** | It could be made clearer there was an introductory animation, by introducing a link at the top of the welcome screen, possibly with a video/animation icon.  The script could be condensed or hidden in a collapsible block.  Professionals: As with the interviewees, many had missed the introductory animation, and it was suggested this could be better signposted, for example with a screenshot or icon at the top of the screen (rather than the bottom). Professionals were even keener for the animation script to be reduced or hidden.  Another new suggestion was for subtitles to be added, and to make it clear that headphones were needed. | Add ‘watch video’ on welcome screen, and launch in ‘modal’ (i.e. self-contained box)  Animation script on ‘How can this help me?’ to be placed in a collapsible block, with ‘read more’ link. Add a subtitles option. |
| **User pathway** | There were some reservations about using the package with others, and another user pathway was proposed – a ‘mood common room’ where the young person and parent/carer could use the package together. A ‘teacher area’ was also proposed.  A few asked for a ‘free text’ box to be added to the initial questions, as a form of diary, and possibly links to messages as the person answered the questions. The mood chart could also be better signposted. Reminders were suggested by many for the mood monitor and the other ‘app’ components.  Professionals: Although it was not an issue raised by the young people, a few professionals had difficulties operating the initial questions/mood monitor. They suggested there could be more explanation for this and for the scores, and more specific guidance on how to help with each aspect, such as mood and enjoyment. Professionals agreed that reminders would be helpful. | Explore how to improve functionality of the initial questions, e.g. progress indicators should not appear ‘clickable’, and add instruction to drag mood indicator.  Explore feasibility of ‘mood common room’.  Add ‘free text’ option.  Add reminders.  Add ‘skip this’ if users do not wish to answer questions. |
| **Dashboard/profile section** | On the dashboard/profile section, some considered the teal colour a little overwhelming for the profile page, and it was suggested there could be more variation and images (possibly the user could choose a colour).  The head icons for the scores could be linked more strongly with suggestions for help; some dashboard messages could be reworded (for example to be less alarmist). | Separate out the subsections within the profile section to make it appear more interesting.  Explore how to link the recommendations with the scores for mood, enjoyment etc. |
| **Tailoring**  **/personalising** | Participants also suggested that the package could be tailored and personalised further, for example in the presentation of subsections/content on the dashboard (also noted by professionals), and by giving more specific guidance in help sections. Workbooks or worksheets were also proposed. A search engine was also suggested. | To change colour of highlighted subsection titles & explore tailoring /search engine options. |
| **Section/Content issues** | | |
| **Information within sections** | There were specific additions suggested for the content, for example there could be more information on CAMHS, treatments and other conditions (although the program was mainly for depression), and on how to help friends who were experiencing difficulties. There were concerns that the package might cause unnecessary worry, and that some would not want to think about their difficulties. The tone and information therefore needed to be accurate, proportional and engaging – and some titles needed rewording e.g. ‘think positively’.  Professionals: It was suggested there could be more information on bereavement, loss and trauma, and links to local resources/services and other resources. There was a call for the use of the term ‘carers’ throughout, and for some of personal stories to be from their perspective. | More information will be added on these issues, and wording to be reviewed.  The term ‘carers’ will be used more frequently, and personal stories will be added from their perspective. |
| **Structure of sections** | The main possible design change related to the restructuring of the sections. Whilst many young people preferred the current approach, especially on mobile devices, others stated the sections should be spliced into separate subsections. Some suggested the text could be cut down a little, for example with the use of bullet points.  Professionals were stronger in their opinion that the sections should be divided into separate screens/subsections, rather than as one scrollable unit, although again there was no consensus. They commented far more than the young people on the excessive blocks of text, particularly in the introductory subsections and personal stories, although this view was based in part on using the package on old operating systems. | The text will be reviewed, and cut down where possible.  The company will review the text size throughout. |
| **Personal stories** | Young people suggested the personal stories would need to be reviewed to ensure it was at the level and using age-appropriate terminology.  Professionals: They called for a more visual approach, especially for the personal stories, through comic strips, ‘student art’ or photographs/videos. Worksheets, printed resources and elements of gamification were also suggested. There may also be a contrast issue with black and red/pink in the family/friends section. | Young people, and an author for young people, will be asked to review the personal stories.  The company will review the colour contrast throughout the package.  Explore adding more visuals. |
| **‘App' and technical issues** | | |
| **Stuff I like/goal setting** | The ‘stuff I like’ and ‘goal setting’ sections could be developed further, with more explanation, colour and engaging aspects, such as images/icons to represent links. | Explore how to make these more engaging e.g. colour/images. |
| **Language** | The ‘app’ would also need to be translated into Welsh, to be consistent with the rest of the package. | The ‘app’ will be translated into Welsh. |
| **Technical issues** | To address issues where giving access to the user to the ‘app’, and related to compatibility with devices and operating systems.  Some found it difficult to access the internet, and wondered whether some information could be stored within the program. The package was slow to load.  Professionals: Program was slow, or the animations did not appear – especially on older NHS computers. They wanted to ensure that users would be logged out if they closed the package. | The digital media company will investigate the technical performance, check compatibility with operating systems etc. – and make improvements. |

**Table 2: Further quotes from the interviews and focus group**

| **Key themes** | **Participant quotes** |
| --- | --- |
| **1: Design features** | *Overall I think it’s a really excellent resource... just making that link from the research place and putting it out there (Psychiatrist: female).*  *I thought it was quite easy to use…you can go at your own pace as well which is quite nice (18-year-old female).*  *I really liked it overall… I really liked most things about it (Mother of 15-year-old female).*  *I think it engages the young person straight away…You get a lot of information in a short space of time…It’s fresh I think (School nurse: female).*  *Mother: I quite like the people…Quite generic...*  *Daughter: It doesn’t make it feel as if it’s you that they’re representing… it’s not like a particular person or particular type of person, it’s just a person (18-year-old female & mother).*  *I thought the graphics were really good because they weren’t too old; they weren’t too young, I just thought that they were spot on (Primary mental health worker: female).* |
| **2: Sections, content** | *I didn't expect that amount of information; the depth is incredible (Mother of 17-year-old female).*  *[The personal stories} would really help a young person identify with it in more of a grounded way so that it wasn’t just theoretical (Social worker:*  *male).*  *I like how the sections are all laid out in the same way, so once you know how to navigate through one, you know how to navigate through all of them, it just makes it smooth (14-year-old male).*  *It can help you…start to identify if you do [have depression], if there is actually a problem so you can then go and seek help… It's not like being sad; it's other stuff as well (18-year-old female).*  *I like the fact that it’s got links to other good sites as well so it’s not trying to be all things to all men (Psychiatrist: male).*  *I felt its stance was quite motivational throughout so there was very much a focus on ways in which you can improve (Mother of 15-year-old female).*  *I thought, in terms of the content, I thought it was good, a good holistic approach to it from a parent’s point of view (Mother of 15-year-old female).* |
| **3: Integration, context** | *Son: Get the widest range of coverage and I think the more people who use something like this the better really, and if there’s people out there having issues then if they can get used to something like this it’s brilliant.*  *Mother: I don’t see why there needs to be a barrier…why you should have to go to somebody to suggest you use it (16-year-old male & mother).*  *Having something like that would bring mood and depression into more light…would reduce the stigma people have on it and they wouldn’t feel so bad about talking to other people about it (16-year-old male).*  *I find it really hard to tell people so that’s why I have the diary… If I just showed her on my phone...it’s better because it also saves time in the meeting, instead of spending half an hour explaining (15-year-old female).*  *My worry would be that people might use it quite superficially or lose motivation (Educational psychologist: female).*  *It’s a pre-IT literate professional group…we’ve had the opportunity to play around on it and think actually this is really good…We need to make sure all the professionals who are likely to recommend it have the same opportunity (Psychiatrist: male).* |
