# Supplementary material for: A Digital Intervention for Adolescent Depression (MoodHwb): Mixed Methods Feasibility Evaluation
Source: JMIR Ment Health. 2020 Jul 17;7(7):e14536. doi: 10.2196/14536 (PMC7395255; doi:10.2196/14536)
Supplement: Multimedia Appendix 2 [file mental_v7i7e14536_app2.docx]

**Figure 1: Questionnaire feedback on how helpful the program was for the young person**

** Compared to before using the program*

*Parents’ responses were about their child*

*Ratings range from 1 (lowest/least improved) to 7 (highest/most improved); 4 = no change /made no difference*

**Figure 2: Questionnaire feedback on how helpful the program was for parents/carers**

** Compared to before using the program*

*Ratings range from 1 (lowest/least improved) to 7 (highest/most improved); 4 = no change /made no difference*

**Figure 3: Feedback on the amount of information in the program from young people (above) and parents/carers (below)**

**
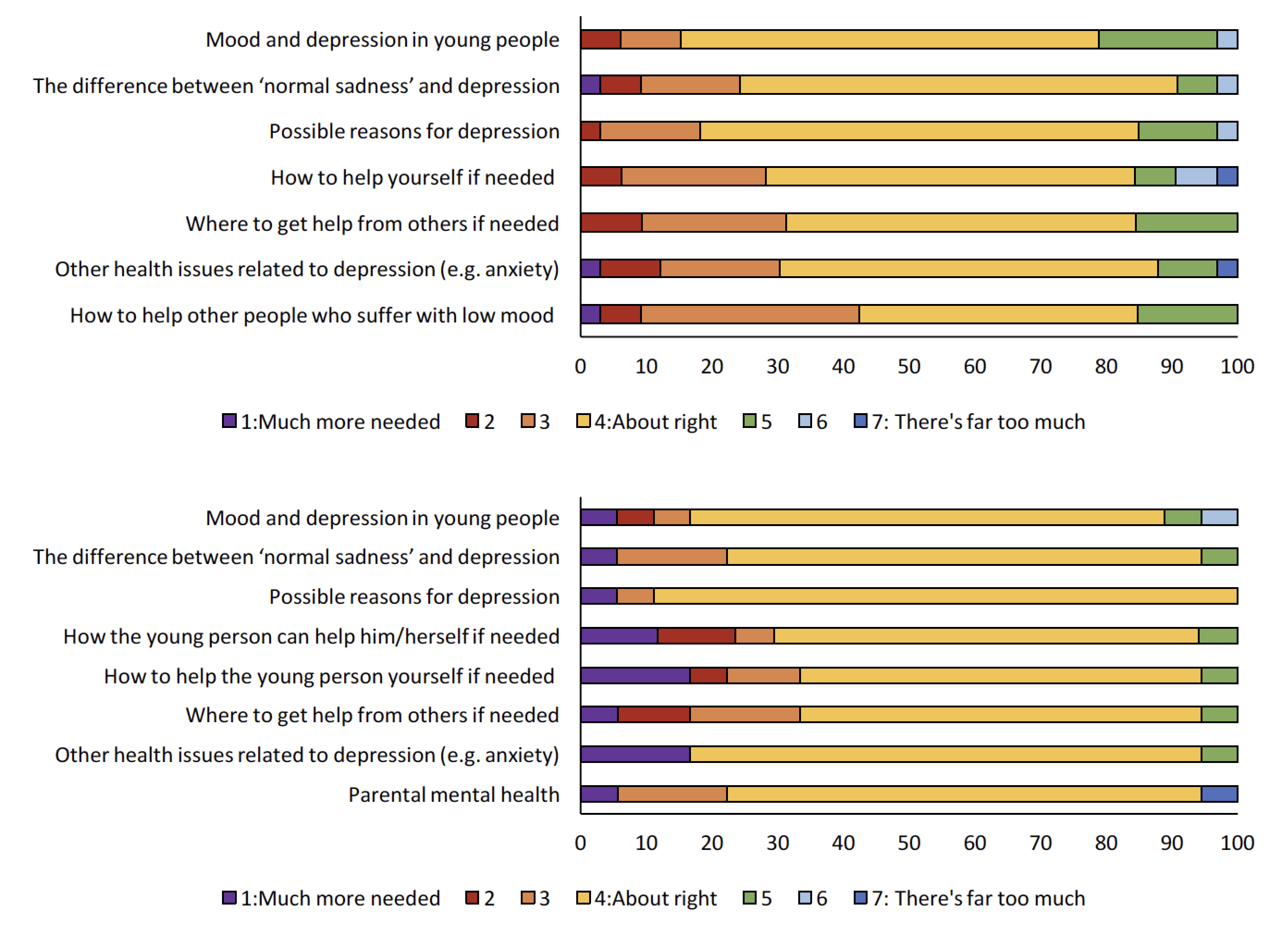
**

**Figure 4: Feedback on design and language**

*Ratings range from 1 (lowest) to 7 (highest)*
